# Social Familiarity Reduces Reaction Times and Enhances Survival of Group-Living Predatory Mites under the Risk of Predation

**DOI:** 10.1371/journal.pone.0043590

**Published:** 2012-08-22

**Authors:** Markus Andreas Strodl, Peter Schausberger

**Affiliations:** Group of Arthropod Ecology and Behavior, Division of Plant Protection, Department of Crop Sciences, University of Natural Resources and Life Sciences, Vienna, Austria; University of Saskatchewan, Canada

## Abstract

**Background:**

Social familiarity, which is based on the ability to recognise familiar conspecific individuals following prior association, may affect all major life activities of group-living animals such as foraging, reproduction and anti-predator behaviours. A scarcely experimentally tested explanation why social familiarity is beneficial for group-living animals is provided by limited attention theory. Limited attention theory postulates that focusing on a given task, such as inspection and assessment of unfamiliar group members, has cognitive and associated physiological and behavioural costs with respect to the attention paid to other tasks, such as anti-predator vigilance and response. Accordingly, we hypothesised that social familiarity enhances the anti-predator success of group-living predatory mites, *Phytoseiulus persimilis,* confronted with an intraguild predator, the predatory mite *Amblyseius andersoni*.

**Methodology/Principal Findings:**

We videotaped and analysed the response of two *P. persimilis* larvae, held in familiar or unfamiliar pairs, to attacks by a gravid *A. andersoni* female, using the behavioural analyses software EthoVision Pro®. Familiar larvae were more frequently close together, reacted more quickly to predator attacks, survived more predator encounters and survived longer than unfamiliar larvae.

**Significance:**

In line with the predictions of limited attention theory, we suggest that social familiarity improves anti-predator behaviours because it allows prey to shift attention to other tasks rather than group member assessment.

## Introduction

Predation is a major selective force shaping the behaviour of prey [1], [2]. To enhance survival under predation risk, animals evolved various behavioural anti-predator mechanisms such as crypsis, defensive or fleeing behaviours, or grouping together to enhance dilution or collective vigilance [3], [4]. Group-living may have partly evolved to reduce predation risk because solitary animals have a relatively limited ability to process multiple information and perform multiple tasks simultaneously, e.g. foraging and anti-predator vigilance [3], [5], [6]–[11]. The more individuals participate in vigilance, the more efficient potential predators can be detected and the less time and energy individual group members have to invest in vigilance [1], [2], [12]. Vigilance may be adjusted to the degree of predation risk, group size and composition, or experience [1], [2], [4], [12]–[16]. For example, group-living animals commonly spend less time being vigilant and invest more time in foraging with increasing group size [1], [2], [12].

Within groups, individuals are expected to position themselves such to maximize their own survival chance under the risk of predation, which depends on the relationships and interactions with other group members [3]. Within-group arrangement is usually non-random and may be influenced by life-stage, age, kinship, size, dominance hierarchy, sex, or social familiarity [3], [16]. We here focused on social familiarity, which requires the ability to discriminate familiar and unfamiliar individuals based on prior association [17]. Many group-living animals of diverse taxa preferentially associate with familiar individuals, which may have positive effects on foraging, life history traits or survival ([18], [19], authors unpublished). Possible cognitive implications of social familiarity are indicated by limited attention theory [7], which postulates that focusing on a given task has cognitive and associated physiological and behavioural costs with respect to the attention paid to other tasks. Familiar individuals usually require less attention than unfamiliar ones [19]–[23] and assorting with familiar individuals should thus lead to increased efficiency in other tasks such as anti-predator behaviour [20], [21]. This has, for example, been shown for groups of familiar trout, which responded more quickly to simulated predator attacks and had higher feeding rates than groups of unfamiliar trout [21]. Studies demonstrating attention shifts induced by social familiarity and its adaptive significance, i.e. its effects on survival or reproduction, under the risk of predation are lacking.

We investigated the influence of social familiarity on anti-predator behaviour of larvae of the group-living predatory mite *Phytoseiulus persimilis* threatened by the intraguild predator *Amblyseius andersoni*. Both predatory mite species live on plants and commonly belong to the same predator guild, sharing the herbivorous two-spotted spider mite, *Tetranychus urticae*, as prey (e.g. [24], [25]). *P. persimilis* is specialized on spider mite prey and lives in groups inside the spider mite patches, while *A. andersoni* is a diet-generalist poorly adapted to exploit tetranychid mites [24]. *P. persimilis*’ development proceeds from the egg to larva, protonymph, deutonymph and the adult [26]. The larvae are six-legged, non-feeding and little mobile whereas later life stages are eight-legged [26]. Due to their limited mobility and small size (∼0.2 mm long), *P. persimilis* larvae are highly vulnerable to intraguild predation by the large gravid *A. andersoni* females (∼0.4 mm long) (e.g. [25]). Both *A. andersoni* and *P. persimilis* are eyeless and sense the environment predominantly by contact and volatile chemosensory cues [27]. Accordingly, *P. persimilis* is able to perceive and respond to chemical cues of the intraguild predator *A. andersoni* with and without physical predator presence [28], [29]. Previous studies revealed that juvenile and adult *P. persimilis*, including larvae, are able to discriminate familiar from unfamiliar individuals, and that social familiarity modulates within-group association, aggression, reproduction and foraging [30]–[33]. Larvae tend to aggregate and remain rather immobile until moulting into protonymphs. Here, we hypothesized that social familiarity adaptively modulates the anti-predator response of group-living larvae by, for example, reducing their reaction time, which is a common indicator of attention (e.g. [21], [34]), and ultimately increases their survival probabilities under the risk of predation.

## Results

**Figure 1:**
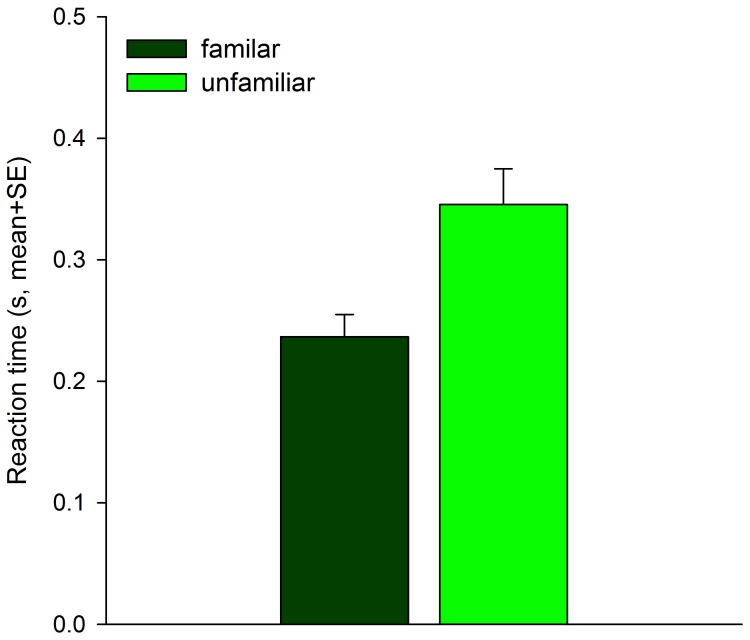
Reaction time of prey larvae to predator encounters. Mean reaction time of *P. persimilis* larvae held in familiar or unfamiliar pairs and threatened by a gravid intraguild predator female of *A. andersoni*.

**Figure 2:**
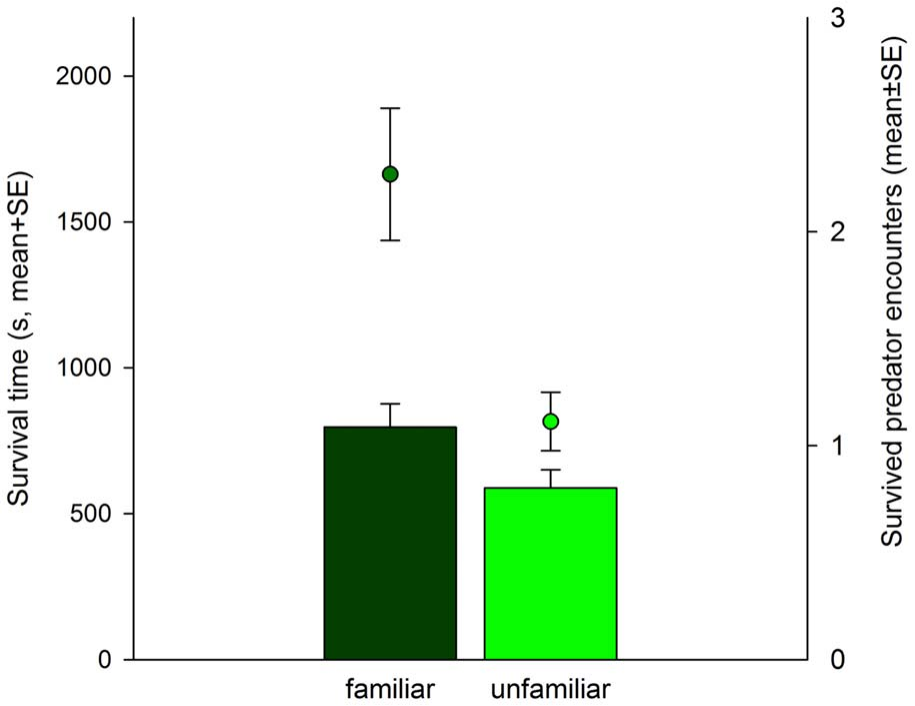
Survival time of prey larvae and survived predator encounters. Mean survival time (left y-axis, bars) and number of predator encounters survived (right y-axis, symbols) of *P. persimilis* larvae held in familiar or unfamiliar pairs and threatened by a gravid intraguild predator female of *A. andersoni.*

Familiar *P. persimilis* larvae reacted more quickly to predator attacks (Wald-*χ^2^_1_* = 5.011, *p* = 0.025), survived more predator encounters (Wald-*χ^2^_1_* = 10.480, *p* = 0.001) and survived longer (Wald-*χ^2^*_1_ = 4.298, *p* = 0.038) than unfamiliar larvae did (Fig. 1, Fig. 2). As a consequence of surviving longer, familiar larvae covered longer distances (mm, mean±SE; familiar: 16.48±2.51, unfamiliar: 9.37±1.33; Wald-*χ^2^*_1_ = 5.996, *p* = 0.014) and moved longer than unfamiliar larvae did (s, mean±SE; familiar: 65.62±3.19, unfamiliar: 51.66±3.85; Wald-*χ^2^*_1_ = 7.484, *p* = 0.006). Unfamiliar and familiar larvae did not differ in velocity (mm/s, mean±SE; familiar: 0.27±0.08, unfamiliar: 0.23±0.02; Wald-*χ^2^*_1_ = 2.233, *p* = 0.135), absolute angular velocity (°/mm, mean±SE; familiar: 49.16±14.33, unfamiliar: 25.78±5.57; Wald-*χ^2^*_1_ = 1.389, *p* = 0.239), absolute meandering (°/mm, mean±SE; familiar: 26.88±6.09, unfamiliar: 65.68±27.46; Wald-*χ^2^*_1_ = 1.939, *p* = 0.146;) and absolute turning angles (Wald-*χ^2^*_1_ = 3.004, *p* = 0.083;°, mean±SE; familiar: 21.05±3.65, unfamiliar: 11.55±1.86). Inter-individual distances of familiar and unfamiliar pairs of larvae were similar (mm, mean±SE; familiar: 6.19±0.46, unfamiliar: 7.21±0.44; t-test: t_78_ = −1.592, *p* = 0.115) but relative proximity, i.e. the percentage of time being close together, was higher in familiar (n = 43) than unfamiliar (n = 46) larvae (%, grouped median; familiar: 0.152, unfamiliar: 0.072; Mann-Whitney-U-test: Z = −2.0, *p* = 0.045).

## Discussion

Fleeing is a common response to avoid predation [2], [13], [35]. Whether fleeing is successful is largely determined by reaction distance, encounter direction, reaction time and escape trajectory, but may also be influenced by other factors such as experience and group characteristics [4], [13], [35]. In our experiment, familiar *P. persimilis* larvae spent more time aggregated (indicated by relative proximity), reacted more quickly to attacks of their predator *A. andersoni*, and thus survived more predator encounters than unfamiliar larvae. The shorter reaction times suggest that social familiarity allowed the mites to shift attention from attention-demanding neighbour assessment to anti-predator vigilance, in accordance with limited attention theory [7], [8], [21], and ultimately enhanced their survival under predation risk.

Thus far, the impact of social familiarity on anti-predator success has been predominantly demonstrated in fish [15]. One apparent phenomenon in group-living fish is that shoals of familiar individuals are more cohesive, indicating that familiarity stabilises shoal structure. For example, stronger cohesion in minnow shoals reduced the capture success of attacking predators, presumably due to enhanced predator confusion [20]. Moreover, individuals within familiar shoals performed typical anti-predator manoeuvres such as dashing or freezing more efficiently than individuals within unfamiliar shoals [20]. Along the same line, Griffiths et al. [21] demonstrated that individuals within familiar groups of brown trout reacted more quickly to simulated predator attacks than individuals within unfamiliar groups. Social familiarity reduced the aggressive interactions between group members and thus enabled the trout to switch attention from intraspecific aggression to anti-predator vigilance [21]. Our study provides evidence that similar phenomena may occur and similar mechanisms may operate in arthropods such as the predatory mite *P. persimilis*.

In related studies we observed the ability of different life stages of *P. persimilis* to preferentially associate with familiar individuals [33]. Here, we show that social familiarity enhances the anti-predator response of larvae. The fragile larvae are often aggregated, possibly to reduce the risk of heterospecific predation and cannibalism [36]. In general, all life stages (egg, larva, protonymph, deutonymph, adult) of *P. persimilis* live together in the ephemeral patches of its preferred prey, the spider mite *T. urticae* [37], [38]. All developmentally more advanced life stages are potential cannibals of larvae [36] and the prey patches are often shared with potential intraguild predators [25]. Thus, the larvae may be often exposed to predator cues and repeatedly encounter potential predators within a patch but not every encounter is life-threatening. Deciding if and when to respond to predator cues involves trade-offs between the benefit of avoiding predator attacks and a variety of costs including energy loss [4], [13]. The costs of fleeing may be loss of energy needed for development or leaving a site with favorable abiotic conditions or possibly attracting the attention of predators due to leaving a safe group formation. Thus, mechanisms optimizing the response of the larvae to a predator such as social familiarity should be selected for [4], [13]. Our study shows that social familiarity provides an adaptive advantage, because larvae held in familiar groups reacted faster to approaching predators than unfamiliar larvae did, which ultimately conferred a fitness gain through longer survival times.

## Materials and Methods

### Origin and Rearing of Experimental Animals

Experimental animals were offspring from females withdrawn from laboratory-reared populations of *P. persimilis* and *A. andersoni*, originally founded with specimens field-collected in Trapani, Sicily [25]. Both species were maintained separately on artificial rearing units each consisting of a plastic tile placed on a water-saturated foam cube (13×13×4 cm) in a plastic box (20×20×5 cm) half-filled with tap water. The edges of the tile were covered by water-saturated tissue paper. *P. persimilis* was fed mixed life-stages of *T. urticae*, reared on whole bean plants *Phaseolus vulgaris*, by adding detached spider mite-infested bean leaves onto arenas in 3 to 4 day intervals. *A. andersoni* was fed mixed life-stages of *T. urticae* brushed from infested bean leaves onto the rearing arenas. Rearing and experimental arenas were stored at 25±1°C, 60±5% relative humidity and 16∶8 h light:dark.

### Generating Familiar and Unfamiliar *P. persimilis* Larvae

Oviposition arenas used to obtain similarly aged *P. persimilis* eggs consisted of single bean leaves (∼60 cm^2^) placed adaxial surface down on a water-saturated, filter paper-covered foam cube (13×13×4 cm) in a plastic box (20×20×5 cm) half-filled with tap water. Strips of moist tissue paper were folded over the edges of the leaves to prevent the mites from escaping. Before placing 40 to 70 *P. persimilis* females for oviposition onto arenas, mixed life-stages of *T. urticae* were brushed from infested bean leaves onto arenas to serve as prey for the predators. *P. persimilis* females were randomly chosen from the laboratory-reared population and eggs were collected after ∼4 h.

*P. persimilis* larvae were familiarised in artificial cages each consisting of a circular cavity (1.5 cm diameter) drilled in an acrylic plate (0.3 cm thick). The cages were covered by gauze on the lower side and on the upper side by a removable microscope slide held in place by a metal clamp [39]. Six *P. persimilis* eggs, randomly taken from the oviposition arenas, were placed together in such a familiarisation cage, where the larvae were allowed to familiarise for ∼8 h after hatching [33]. All larvae were derived from the same group of ovipositing females and had thus the same degree of genetic relatedness to each other. The only difference between familiar and unfamiliar larvae was inter-larvae familiarity or not generated in the familiarisation cages.

### Experimental Procedure

To start the experiment, either two familiar or two unfamiliar *P. persimilis* larvae were placed in the centre of a circular experimental arena. Before the experiment the two larvae of a pair were marked with differently sized black water colour dots using a fine brush on their dorsal shields to make them distinguishable for the tracking module of the behavioural analyses software EthoVision Pro®. Familiar pairs were taken out of the same familiarisation cage, whereas larvae of unfamiliar pairs were randomly taken out of two different familiarisation cages using a fine brush. Experimental arenas were flat polypropylene discs (1.4 cm diameter) floating on the surface of a water column in an acrylic cylinder (height 2.0 cm, inner diameter 1.6 cm), leaving a ∼0.1 cm water barrier between the inner margin of the cylinder and the edge of the disc, preventing the mites from escaping. After ∼10 min acclimatisation of the larvae to the novel environment, a single gravid *A. andersoni* female, starved for 20 h before the experiment, was released in the centre of the arena and videotaping was started. The *A. andersoni* females were starved to increase the likelihood of attack on *P. persimilis* larvae. The experiment ended when a larva was killed by *A. andersoni* or died in the water barrier. 49 familiar and 49 unfamiliar pairs of larvae were tested. Each disc, each *P. persimilis* larva and each *A. andersoni* female was used only once. Replicates with familiar and unfamiliar larvae were videotaped at similar daytimes in an air-conditioned room without natural light.

### Behavioural Analyses

The behaviour of the mites was videotaped with a Leica IC-A video module integrated in a Leica M50 microscope. To digitalise the videos, the video module was interfaced by the frame grabber HaSoTec FG-33-II (Indeo codec). EthoVision Pro® 3.1 [40] was used to analyse the behaviour of the larvae. The subtraction method was used for object detection and discrimination. To prevent electronic noise from being scored as genuine movement of the larvae by EthoVision Pro® [40], the calculation settings of the analysis module were adjusted to a minimum distance of 0.2 mm, which corresponds approximately to one larval body length, and a minimum velocity of 0.1 mm/s. Using EthoVision Pro® we analysed the inter-individual distances (mm), velocity (mm/s), distance moved (mm), moving time (s), absolute turning angle (°), absolute angular velocity (°/s), absolute meandering (°/mm) and relative proximity of the larvae. The absolute turning angle equals the average direction of an individual’s movement during one sample relative to the average direction of movement during the consecutive sample (sample rate: 5 samples/s). The absolute angular velocity (°/s) is calculated by dividing the turning angle by the sample interval and is an indicator of how fast an object is changing its direction. Meandering is the interrelation of the direction of an individual’s movement (turning angle) and the distance moved indicating the path tortuosity of the experimental organism. Relative proximity is a state parameter representing the percentage of time where two experimental organisms are together within a predefined distance. The “in proximity”-distance was predefined as two larval body lengths (∼0.4 mm). 43 familiar and 46 unfamiliar pairs out of 49 each videotaped could be successfully tracked and analysed with EthoVision Pro®. Personal video analysis additionally allowed for recording survival time (s), the number of encounters with *A. andersoni* survived and the reaction time of each larva when encountered by the predator. The predator was considered to encounter the larva when it came within a distance of approximately one larval body length (∼0.2 mm) to the larva. Larvae were considered to react to encounters with *A. andersoni* when they moved away from the predator after encounter. The reaction time was estimated by counting the number of single video frames between the encounter of the predator and the first observable movement of the larva away from the predator. For analysis, we only used the first two encounters of *A. andersoni* with each larva, within the first 10 minutes of each replicate, to avoid any obscuring effect of learning possibly caused by differing numbers of survived encounters. The videos were recorded with 25 progressive frames per second (PAL colour encoding system), which allowed to transform the number of frames into seconds.

### Statistical Analyses

All statistical analyses were performed using SPSS 15.0.1 for Windows (SPSS Inc., Chicago, IL, USA, 2006). Separate generalized estimating equations (GEE) with normal distribution, identity link and autocorrelation structure between the two larvae of a pair were used to test the effects of familiarity on time, distance and path shape parameters within larval pairs. The reaction times were log-transformed before statistical analysis to normalize the data. The inter-individual distances between familiar and unfamiliar larvae were compared using t-tests for independent samples. Mann-Whitney U test was used to compare the proximity parameters within familiar and unfamiliar larvae due to non-normality.
